# Effect of Hydrophobic Acrylic versus Hydrophilic Acrylic Intraocular Lens on Posterior Capsule Opacification: Meta-Analysis

**DOI:** 10.1371/journal.pone.0077864

**Published:** 2013-11-05

**Authors:** Ying Li, Jiaxing Wang, Zhuo Chen, Xin Tang

**Affiliations:** 1 Clinical College of Ophthalmology, Tianjin Medical University, Tianjin Eye Hospital, Tianjin, China; 2 Department of Ophthalmology, Tianjin Medical University General Hospital, Tianjin, China; University Hospital Heidelberg, Germany

## Abstract

**Purpose:**

This meta-analysis aims to evaluate the differences in performance of posterior capsular opacification (PCO) between hydrophobic acrylic intraocular lens (IOLs) and hydrophilic acrylic IOLs.

**Setting:**

Tianjin Medical University, Tianjin Eye Hospital, Tianjin Key Laboratory of Ophthalmology and Visual Science, Tianjin, China.

**Design:**

Systematic review of randomized controlled trials (RCTs) or meta-analysis.

**Methods:**

An electronic literature search was performed using the PubMed, EMBASE and Cochrane Library database before May in 2013 to identify prospective RCTs comparing hydrophobic acrylic IOLs and hydrophilic acrylic IOLs in patients after phacoemulsification with IOL implantation with a follow-up time of at least 1 year. Pertinent studies were selected by meeting predefined criteria and reviewed systematically by meta-analysis. The PCO scores and YAG capsulotomy rate, as indicator of PCO, were measured and discussed in a meta-analysis. Standardized mean differences (SMD), relative risk ratio (RR), and the pooled estimates were computed according to a random effect model or fixed effect model.

**Results:**

Nine prospective RCTs involving 861 eyes were included in the current meta-analysis. The hydrophobic acrylic IOLs were favored and the pooled SMD of PCO severity was1.72 (95% confidence interval (CI), 0.20 to 1.23, *P* = 0.0002) and 1.79 (95% CI, 0.95 to 2.64, *P*<0.0001) with 1-year follow-up and 2-year follow-up respectively. The pooled RR of Nd:YAG laser capsulotomy rates at postoperative 2-year follow-up was 6.96 (95% CI, 3.69 to 13.11, *P*<0.00001) comparing hydrophilic acrylic IOLs with hydrophobic acrylic IOLs.

**Conclusions:**

Compared with hydrophilic acrylic IOLs, the hydrophobic acrylic IOLs showed superior reduction in rates of PCO and laser capsulotomy in 2-year follow-up. More RCTs with standard methods for longer follow-up are needed to validate the association.

## Introduction

With the development of surgical techniques and biomaterial science, cataract surgery with intraocular lens (IOL) implantation has brought great benefits for patients. However, posterior capsule opacification (PCO), remains the most frequent long-term complication [1], decreasing the visual performance in 1 or 2 years after cataract surgery. Although treatment with Nd:YAG laser capsulotomy is effective, the complications, such as retina detachment, macular edema, intraocular pressure elevation [2], cannot be ignored.

Intraocular lens, with various designs and materials, have been observed in playing a vital role in the developmentof PCO. Two areas of concern are the biomaterials and the edge design of IOLs. Studies have shown that the rate of PCO with sharp edge designed IOLs was lower due to the inhibition of lens epithelial cells (LECs) migration [3], [4]. Acrylic IOLs with hydrophilic or hydrophobic surfaces, as two types of biocompatibility materials, safe for intraocular implantation, have a long history of clinical practice and have shown significantly lower rates of PCO and less Nd:YAG laser capsulotomy [5]–[8]. Studies found that acrylic material has a relatively low propensity to induce cell proliferation in the capsular bag [9]. Yet whether the hydrophilic or hydrophobic IOLs are better for PCO prevention remains controversial.

Numerous studies have compared on PCO to different designs and materials combinations of IOLs. Few comparative studies, however have evaluated the differences between hydrophobic acrylic IOLs and hydrophilic acrylic IOLs with the same edge design specifically. The aim of this meta-analysis is to investigate the differences between hydrophobic and hydrophilic IOLs with the same edge design in the development of PCO and the rate of Nd:YAG laser capsulotomy in a 2 year period.

## Materials and Methods

### Literature Search

**Figure 1:**
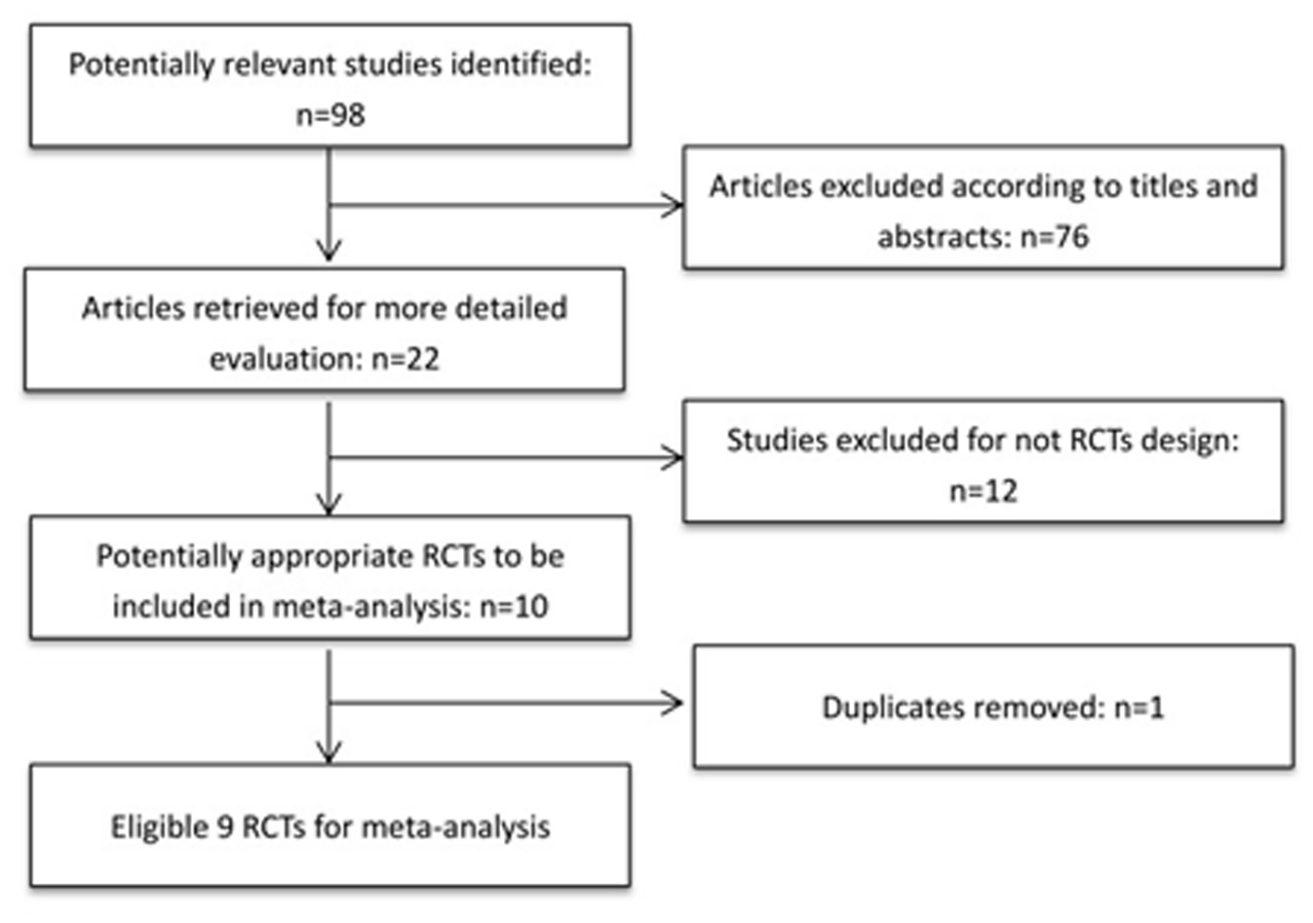
Flow chart of literature search and study selection.

This review was conducted following the QUOROM guideline standards [10]. Reports of randomized controlled trials (RCTs) comparing hydrophobic acrylic and hydrophilic acrylic IOLs in patients after phacoemulsification with IOL implantation were identified through a computerized literature search. The systematic search was conducted in the PubMed, EMBASE, and Cochrane Controlled Trials Register database up to the end of May 2013 by using the search terms “*posterior capsular opacification*” “*intraocular lens*” “*hydrophilic*” “*hydrophobic*” and limiting the search to reports of randomized controlled trials. The abstract of all potentially relevant articles were screened to determine their relevance followed by evaluation of candidate full articles. In addition, literature reference proceedings were scanned manually to obtain extra eligible trials until no more relevant trials were found in databases. For data collected from duplicate patient groups, only the most recent studies were included in each part of meta-analysis. The process of trials selection is shown in Figure 1. Two independent investigators performed the literature search (YL, JXW).

### Selection Criteria

Selected trials fulfilling the following inclusion criteria were used in this analysis: (1) prospective design, randomized controlled trial; (2) population, patients with senile cataract undergoing cataract surgery; (3) intervention, phacoemulsification and IOL implantation; (4) comparison, hydrophobic acrylic and hydrophilic acrylic IOLs; all involved IOLs are designed with sharp edge;(5) outcome variables, at least one of the following primary outcome variables: PCO score, Nd:YAG capsulotomy rate.

Exclusion criteria were as follows: (1) patients with a history of ocular diseases other than senile cataract; (2) patients with a history of intraocular surgery other than IOL; (3) Cases with intraoperative complications, such as incomplete continuous curvilinear capsulorhexis (CCC), posterior capsule rupture or whose with postoperative complications (eg, iris synechia); (4) follow-up time of less than one year.

### Data Extraction and Quality Assessment

Two reviewers (YL, JXW) completed searches independently according to the above the criteria, assessed the methodological quality of trials and extracted data from each eligible randomized clinical trial results. Differences were resolved by discussion to reach consensus between the investigators and results were checked by the third author (ZC) when required. The following items were collected from each trial: author's name, year of publication, design of study, patients' mean age, gender, group size, IOL biomaterials and special designs, evaluation of PCO, and Nd:YAG capsulotomy (number or rate), follow-up period, respectively.

### Outcome Measures

The primary outcome measure was the difference in PCO intensity or PCO score from software between the 2 types of IOLs at 1-year and 2-year follow-up post-operation. The secondary outcome measures were Nd:YAG capsulotomy rate at 2-year follow-up post-operation. For those patients who underwent Nd:YAG capsulotomy, the PCO value just before Nd:YAG capsulotomy was used for further statistical analysis. If there was more than 1 published report on the same population or group of patients, the most recent results with complementary data from previous articles were used for statistical analysis.

### Statistical Analysis and Assessment of Heterogeneity

According to various measurement scales, PCO severity outcomes, as continuous data, were pooled using standardized mean differences (SMD) with 95% confidence intervals (CIs)[11]. The data of Nd:YAG capsulotomy rate, as dichotomous data, were tabulated into 2×2 tables and the relative risk ratio (RR) and 95% CIs of the results were compared. Heterogeneity was also assessed through chisquare test, and an *I* ^2^ value greater than 50%, *P*<0.1 was considered significant. We evaluated the pooled summary effect by using random-effect model. Otherwise(*I* ^2^ value≤50%, *P*≥0.1), data were combined using fixed-effect model to reduce the heterogeneity between studies. Finally, publication bias was assessed visually with funnel plots. The statistical analyses were carried out with RevMan software (version 5.0, The Cochrane Collaboration).

## Results

### Study included

The results of our research strategy are shown in Fig. 1. A total of 132 potentially relevant publications were identified through the literature search from multiple databases before May in 2013, 20 of which were based on their titles and abstracts. Only 9 random controlled trials [2], [8], [12]–[18] were eligible for this meta-analysis.

### Quality assessment of eligible studies and characteristics of included IOL

The methodological quality of trials that were identified and the characteristics of 9 potential RCTs included in the current meta-analysis are presented in Table 1. In these 9 RCTs included in this meta-analysis, the investigators described the random component in a sequence generation process such as: using an envelope [2], [12], [17], or referring to a computer random number generator[14], [15]. Only 1 of 9 studies used double-blinding^12^, while the others used single-blinding[2], [8], [13]–[18]. Recruited RCTs were carried out in many countries including United Kingdom, Japan, India, Sweden and Korea. The length of the studies was between 1 and 2 years. All studies included described the dropout patients' number and reasons respectively. Characteristics of IOLs included in the recruited studies are presented in Table 2.

**Table 1:** Evaluation of the quality of RCTs included in the meta-analysis.

Study	Random	Blind	Withdraw	Jadad score(0–5)
Gangwani 2011	Appropriate	Double-blind	Described	5
Iwase 2011	Yes	Yes	Described	3
Vasavada 2011	Appropriate	Yes	Described	4
Kang 2009	Yes	Yes	Described	3
Cleary 2009	Appropriate	Yes	Described	4
Kugelberg 2008	Appropriate	Yes	Described	4
Hancox 2007	Yes	Yes	Described	3
Kugelberg 2006	Appropriate	Yes	Described	4
Heatley 2005	Yes	Yes	Described	3

**Table 2:** Characteristics of IOLs included in the meta-analysis.

IOLs	Optic Material	Haptic Material	Lens Type	Optic Diameter	Sharp Edge Design	Optic Shape	Haptic Angulation	Distinctive Feature	PCO evaluation system
Acrysof SA60AT	Hydrophobic Acrylic	Acrylic	1-piece	6 mm	Posterior	Anterior Asymmetric biconvex	0	N/A	AQUA Scheimpflug POCOman
Acrysof SN60WF	Hydrophobic Acrylic	Acrylic	1-piece	6 mm	Posterior	Posterior biconvex	0	N/A	EPCO
Sensar AR40e	Hydrophobic Acrylic	PMMA	3-piece	6 mm	Posterior	Equal biconvex	5	N/A	POCOman
Acrysof MA60AC	Hydrophobic Acrylic	PMMA	3-piece	6 mm	Posterior	Anterior Asymmetric Biconvex	10	N/A	POCOman
Acrysof MA30AC	Hydrophobic Acrylic	PMMA	3-piece	5.5 mm	Double	Anterior Asymmetric Biconvex	5	N/A	POCOman
Idea 613XC	Hydrophilic Acrylic	Acrylic	1-piece	6 mm	Double	Biconvex	9	Broad optic-haptic junction	AQUA
Meridian HP60M	Hydrophilic Acrylic	PMMA	1-piece	6 mm	Double	Anterior biconvex	6	N/A	Scheimpflug
C-flex 570C	Hydrophilic Acrylic	Acrylic	1-piece	5.75 mm	Double	N/A	0	N/A	EPCO
Akreos Adapt	Hydrophilic Acrylic	Acrylic	1-piece	6 mm	Double	Equal biconvex	0	N/A	EPCO
Bio Vue3	Hydrophilic Acrylic	PVDF+	3-piece	6 mm	Double	Equal biconvex	5	Heparin surface modification	POCOman
MC611M	Hydrophilic Acrylic	Acrylic	1-piece	6 mm	Double	N/A	0	Broad optic-haptic junction	POCOman
BL27	Hydrophilic Acrylic	Acrylic	1-piece	6 mm	Posterior	N/A	0	N/A	POCOman
1CU	Hydrophilic Acrylic	Acrylic	1-piece	5.5 mm	Double	Equal biconvex	0	4 haptics Accommodation	POCOman
Centerflex 570H	Hydrophilic Acrylic	Acrylic	1-piece	5.75 mm	Posterior	Equal biconvex	0	N/A	POCOman

### Efficacy analysis

#### Effects of hydrophobic acrylic versus hydrophilic acrylic IOLs on development of posterior capsule opacification in 1-year follow-up

**Figure 2:**
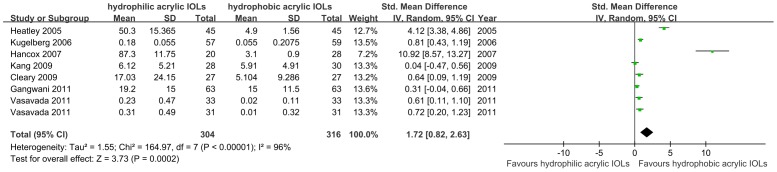
Forest plots describing effects of hydrophobic acrylic versus hydrophilic acrylic IOLs on development of posterior capsule opacification in 1-year follow-up. (Chi^2^  =  chi square statistic, CI  =  confidence interval, df  =  degrees of freedom, I^2^  =  I-square heterogeneity statistic, IV  =  inverse variance, SMD  =  standard mean difference, Z  =  Z-statistic).

Based on 7 studies (620 total eyes) that evaluated PCO after a 1-year follow-up period[2], [8], [12], [14]–[16], [18], hydrophobic acrylic IOLs were associated with significantly lower PCO scores than hydrophilic acrylic IOLs; the SMD was 1.72 (95% CI, 0.82 to 2.63, *P* = 0.0002). The data showed that they had heterogeneity of effect size (*P*<0.00001, *I* ^2^ = 96%), so the random effect model was used for meta-analysis. The results are shown in Figure 2.

#### Effects of hydrophobic acrylic versus hydrophilic acrylic IOLs on development of posterior capsule opacification in 2-year follow-up

**Figure 3:**
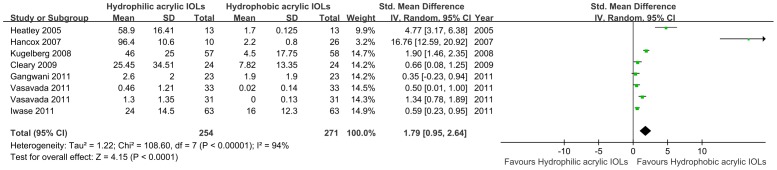
Forest plots describing effects of hydrophobic acrylic versus hydrophilic acrylic IOLs on development of posterior capsule opacification in 2-year follow-up. (Chi^2^  =  chi square statistic, CI  =  confidence interval, df  =  degrees of freedom, I^2^  =  I-square heterogeneity statistic, IV  =  inverse variance, SMD  =  standard mean difference, Z  =  Z-statistic).

Seven studies involving 525 eyes used different scales to report the outcomes for PCO after 2-year follow-up [8], [12]–[14], [16]–[18]. They also had heterogeneity of effect size (*P*<0.00001, *I* ^2^ = 94%), so the random effect model was used for meta-analysis. A significant difference was found between the hydrophobic acrylic and hydrophilic acrylic IOLs; the SMD was 1.79 (95% CI, 0.95 to 2.64, *P*<0.0001), indicating that hydrophobic acrylic IOLs were associated with lower PCO score in 2-year follow-up. The results are shown in Figure 3.

#### Effects of hydrophobic acrylic versus hydrophilic acrylic IOLs on rate of Nd:YAG capsulotomy in 2-year follow-up

**Figure 4:**
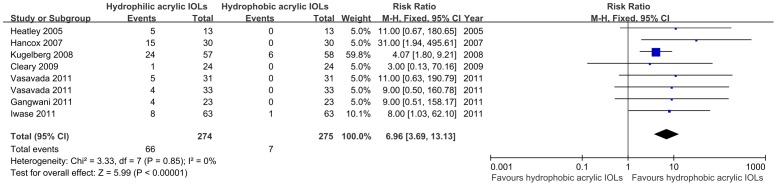
Forest plots describing Effects of hydrophobic acrylic versus hydrophilic acrylic IOLs on rate of Nd:YA Gcapsulotomy in 2-year follow-up. (Chi^2^  =  chi square statistic, CI  =  confidence interval, df  =  degrees of freedom, I^2^  =  I-square heterogeneity statistic, IV  =  inverse variance, RR  =  risk ratio, Z  =  Z-statistic).

Seven studies[8], [12]–[14], [16]–[18] involving 546 eyes compared the Nd:YAG capsulotomy rate of hydrophobic acrylic IOLs with hydrophilic acrylic IOLs in a 2-year follow-up period. No statistical heterogeneity was detected between studies (*P* = 0.85, *I* ^2^ = 0%). Therefore, the fixed effect model was analyzed for this research. The results from analysis suggest that hydrophobic acrylic IOLs had a lower Nd:YAG capsulotomy rate; the RR was 6.96(95% CI, 3.69 to 13.13, *P*<0.00001). The results are shown in Figure 4.

### Publication Bias

The publication bias was assessed graphically for each outcome independently using funnel plots. The funnel shaped with the apex near the symmetry, which suggesting publication bias is less of a concern.

## Discussion

At present, PCO remains the most common complication of modern cataract surgery. There is considerable interest in the impact of IOL on the development of PCO since characteristics and designs of IOLs play a crucial role in preventing PCO. Furthermore, differences in PCO performance between IOLs were likely to reflect their distinction in biomaterials and designs. Both hydrophilic and hydrophobic have been commonly used in cataract surgeries. Hydrophobic acrylic IOLs have a long track record of good PCO rate [19]. However, due to the mechanical properties, hydrophilic acrylic IOLs may be more suitable for implantation through smaller IOL injection systems [12]. Additionally they may have superior biocompatibility and less macrophage adhesion especially when a blood-aqueous barrier breakdown has occurred, such as those with glaucoma, uveitis, and diabetes mellitus [20]. Although higher PCO rates have been reported for hydrophilic materials [2], [17], [21], controversy remains over whether this finding is a result of differences in material properties or variation in the optic edge design [22]. The potential mechanism that IOLs with sharp optic edge prevent PCO, including cell migration prevention [23], pressure atrophy [15] and contact inhibition [24], have gained acceptance widely. The PCO rate in hydrophilic acrylic IOLs implantation, which have an improved 360-degree sharp edge, is reported lower than with older hydrophilic models [25], [26]. It should not be ignored that many studies have a significant limitation, comparing hydrophilic IOLs with round optic edges and hydrophobic IOLs with sharp optic edges [8], [27], [28].

Since complete surgical removal lens epithelial cells (LECs) is not possible with modern surgical technology, the migration of remaining equatorial LECs which form PCO may be difficult to avoid. As a proliferative pathological process, there is a close relationship between the severity of PCO and post-operative follow-up time. The longer follow-up period, the better clinicians understand the effect of IOLs on the development of PCO. Some studies have shown no differences in PCO rates between the two different materials IOLs after cataract surgery 1 year [13], [15]. Others have shown significantly different results [14], [16]. Indeed, some researches have addressed the influence of various IOLs on the incidence of PCO in different lengths of follow-up [1], [29]. Therefore long term randomized controlled trials, especially with a multi center large sample size, are needed to evaluate further effects of various IOL biomaterials with similar optic edge designs in decreasing PCO and Nd:YAG capsulotomy rates.

This meta analysis evaluated the 1-year and 2-year postoperative PCO results and rate of Nd:YAG laser capsulotomy in 2-year follow-up between hydrophilic acrylic and hydrophobic acrylic IOLs implantation respectively. All the IOLs involved in this analysis were designed to prevent PCO by incorporating a sharp edge. Therefore, the differences shown in this analysis may be interpreted primarily based on material effects. The result of this meta-analysis support the theory that compared to hydrophilic acrylic IOLs, hydrophobic acrylic IOLs led to significantly less PCO in 1-year and 2-year follow-up periods. Meanwhile, the rates of Nd:YAG laser capsulotomy were also reduced following hydrophobic acrylic IOLs implantation 2-year post-operation. A reasonable interpretation for the difference in this meta-analysis may be that hydrophobic acrylic IOLs can adhere to collagen membrane [30], leading to tight apposition of IOLs in posterior capsular bag, and advanced adhesiveness through fibronectin [8]. This may result in less space between IOLs and posterior capsule where the LECs could migrate. On the other hand, the hydrophilic surface properties were found to promote proliferation and migration of LECs from the equatorial area to the visual region [31]. Moreover, studies compared electron microscope images and found the edge of hydrophilic IOLs to be less sharp than hydrophobic IOLs at several optical powers [13]. The potential reason could be hydrophilic acrylic IOLs are machined in the dehydrated state and then rehydrated, which can lead to loss of edge sharpness [32]. These differences in manufacturing techniques may explain why hydrophobic IOLs appear to have relatively better PCO performance. The recent study [33] suggest a new aspect to consider in lens material, hybrid technique (hydrophilic center and hydrophobic surface coated IOLs), indicating that hybrid IOLs are less susceptible than hydrophilic IOLs to cell adhesion and less susceptible than hydrophobic IOLs to glistening formation. The copolymer hybrid IOLs may present certain important advantages and should therefore be further evaluated with PCO performance in clinical studies.

The studies in this meta-analysis used a variety of different evaluation systems for PCO analysis, such as Scheimpflug photography system [13], POCOman system [2], [8], [15]–[18], AQUA (Automated Quantification of After-Cataract system) 12, and EPCO (Evaluation of Posterior Capsule Opacification software) [14]. Each of these software systems has particular features that made them vulnerable. POCOman and AQUA system are objective, however, they do not incorporate whether PCO is peripheral or central location and show limited points [29]. EPCO evaluates the construct validity [34], but it is a subjective style. On account of the different analysis systems for PCO across the studies, SMDs were used in this meta-analysis as in previous studies [29].

Limitation of this meta-analysis may be found. First, even though all the IOLs in this meta-analysis were designed with sharp edge, as a key factor in retarding PCO, there were various extra factor could make an impact. For example, the IOLs with broad optic-haptic junctions [16], the heparin-surface-modified [15], the asphericity on the posterior surface [35], the degree of haptic angulation [36], and optic size [37] appear to exert influence upon PCO formation. The pathophysiology of PCO is multifactorial. Since dissociation of each factor in PCO development is almost impossible, it is very difficult to observe individual elements in clinical practice. Second, although we conducted a thorough electronic search and a manual search of the references of relevant results to minimize selection and publication bias, there were not sufficient studies included to verify if asymmetry exists in a funnel plot. Consequently, long-term postoperative follow-up of multicenter large-sample size randomized controlled trials for PCO development after cataract surgery are necessary.

## Supporting Information

Checklist S1 PRISMA 2009 Checklist.
(DOC)

Prisma Flow Diagram S1 PRISMA 2009 Flow Diagram.
(DOC)

## References

[1] Cheng JW, Wei RL, Cai JP, Xi GL, Zhu H, et al (2007) Efficacy of different intraocular lens materials and optic edge designs in preventing posterior capsular opacification: a meta-analysis. Am J Ophthalmol 143: 428–436. 17224119 10.1016/j.ajo.2006.11.045

[2] Kugelberg M, Wejde G, Jayaram H, Zetterström C (2006) Posterior capsule opacification after implantation of a hydrophilic or a hydrophobic acrylic intraocular lens: one-year follow-up. Journal of cataract and refractive surgery 32: 1627–1631. 17010858 10.1016/j.jcrs.2006.05.011

[3] Buehl W, Findl O (2008) Effect of intraocular lens design on posterior capsule opacification. Journal of cataract and refractive surgery 34: 1976–1985. 19006748 10.1016/j.jcrs.2008.07.029

[4] Buehl W, Findl O, Menapace R, Sacu S, Kriechbaum K, et al (2005) Long-term effect of optic edge design in an acrylic intraocular lens on posterior capsule opacification. Journal of cataract and refractive surgery 31: 954–961. 15975461 10.1016/j.jcrs.2004.09.053

[5] Findl O, Menapace R, Sacu S, Buehl W, Rainer G (2005) Effect of optic material on posterior capsule opacification in intraocular lenses with sharp-edge optics: randomized clinical trial. Ophthalmology 112: 67–72. 15629822 10.1016/j.ophtha.2004.07.032

[6] Beltrame G, Salvetat ML, Chizzolini M, Driussi GB, Busatto P, et al (2002) Posterior capsule opacification and Nd:YAG capsulotomy rates after implantation of silicone, hydrogel and soft acrylic intraocular lenses: a two-year follow-up study. European journal of ophthalmology 12: 388–394. 12474921 10.1177/112067210201200508

[7] Hollick EJ, Spalton DJ, Ursell PG, Pande MV, Barman SA, et al. (1999) The effect of polymethylmethacrylate, silicone, and polyacrylic intraocular lenses on posterior capsular opacification 3 years after cataract surgery. Ophthalmology. 106: :49–54; discussion 54–45 10.1016/S0161-6420(99)90047-7 9917780

[8] Heatley CJ, Spalton DJ, Kumar A, Jose R, Boyce J, et al (2005) Comparison of posterior capsule opacification rates between hydrophilic and hydrophobic single-piece acrylic intraocular lenses. Journal of cataract and refractive surgery 31: 718–724. 15899448 10.1016/j.jcrs.2004.08.060

[9] Apple DJ, Peng Q, Visessook N, Werner L, Pandey SK, et al (2001) Eradication of posterior capsule opacification: documentation of a marked decrease in Nd:YAG laser posterior capsulotomy rates noted in an analysis of 5416 pseudophakic human eyes obtained postmortem. Ophthalmology 108: 505–518. 11237905 10.1016/s0161-6420(00)00589-3

[10] Ramos Macias A, de Miguel Martinez I, Martin Sanchez AM, Gómez González JL, Martín Galán A (1992) The incorporation of acyclovir into the treatment of peripheral paralysis. A study of 45 cases. Acta otorrinolaringologica espanola 43: 117–120. 1605959

[11] Zhu XF, Zou HD, Yu YF, Sun Q, Zhao NQ (2012) Comparison of blue light-filtering IOLs and UV light-filtering IOLs for cataract surgery: a meta-analysis. PloS one 7: e33013. 22412976 10.1371/journal.pone.0033013 PMC3296774

[12] Gangwani V, Hirnschall N, Koshy J, Crnej A, Nishi Y, et al (2011) Posterior capsule opacification and capsular bag performance of a microincision intraocular lens. Journal of cataract and refractive surgery 37: 1988–1992. 21907536 10.1016/j.jcrs.2011.05.035

[13] Iwase T, Nishi Y, Oveson BC, Jo YJ (2011) Hydrophobic versus double-square-edged hydrophilic foldable acrylic intraocular lens: effect on posterior capsule opacification. Journal of cataract and refractive surgery 37: 1060–1068. 21596248 10.1016/j.jcrs.2010.12.059

[14] Vasavada AR, Raj SM, Shah A, Shah G, Vasavada V, et al (2011) Comparison of posterior capsule opacification with hydrophobic acrylic and hydrophilic acrylic intraocular lenses. Journal of cataract and refractive surgery 37: 1050–1059. 21596247 10.1016/j.jcrs.2010.12.060

[15] Kang S, Choi JA, Joo CK (2009) Comparison of posterior capsular opacification in heparin-surface-modified hydrophilic acrylic and hydrophobic acrylic intraocular lenses. Japanese journal of ophthalmology 53: 204–208. 19484436 10.1007/s10384-008-0646-3

[16] Cleary G, Spalton DJ, Hancox J, Boyce J, Marshall J (2009) Randomized intraindividual comparison of posterior capsule opacifcation between a microincision intraocular lens and a conventional intraocular lens. Journal of cataract and refractive surgery 35: 265–272. 19185241 10.1016/j.jcrs.2008.10.048

[17] Kugelberg M, Wejde G, Jayaram H, Zetterström C (2008) Two-year follow-up of posterior capsule opacification after implantation of a hydrophilic or hydrophobic acrylic intraocular lens. Acta ophthalmologica 86: 533–536. 18081899 10.1111/j.1600-0420.2007.01094.x

[18] Hancox J, Spalton D, Heatley C, Jayaram H, Yip J, et al (2007) Fellow-eye comparison of posterior capsule opacification rates after implantation of 1CU accommodating and AcrySof MA30 monofocal intraocular lenses. Journal of cataract and refractive surgery 33: 413–417. 17321391 10.1016/j.jcrs.2006.10.056

[19] Leydolt C, Davidovic S, Sacu S, Menapace R, Neumayer T, et al (2007) Long-term effect of 1-piece and 3-piece hydrophobic acrylic intraocular lens on posterior capsule opacification: a randomized trial. Ophthalmology 114: 1663–1669. 17822973 10.1016/j.ophtha.2006.12.016

[20] Richter-Mueksch S, Kahraman G, Amon M, Schild-Burggasser G, Schauersberger J, et al (2007) Uveal and capsular biocompatibility after implantation of sharp-edged hydrophilic acrylic, hydrophobic acrylic, and silicone intraocular lenses in eyes with pseudoexfoliation syndrome. Journal of cataract and refractive surgery 33: 1414–1418. 17662434 10.1016/j.jcrs.2007.05.009

[21] Findl O, Buehl W, Bauer P, Sycha T (2010) Interventions for preventing posterior capsule opacification. Cochrane database of systematic reviews 2: CD003738. 10.1002/14651858.CD003738.pub3 PMC10658648 20166069

[22] Werner L, Tetz M, Feldmann I, Bücker M (2009) Evaluating and defining the sharpness of intraocular lenses: microedge structure of commercially available square-edged hydrophilic intraocular lenses. Journal of cataract and refractive surgery 35: 556–566. 19251151 10.1016/j.jcrs.2008.11.042

[23] Nagamoto T, Fujiwara T (2003) Inhibition of lens epithelial cell migration at the intraocular lens optic edge: role of capsule bending and contact pressure. Journal of cataract and refractive surgery 29: 1605–1612. 12954314 10.1016/s0886-3350(03)00050-6

[24] Nishi O, Yamamoto N, Nishi K, Nishi Y (2007) Contact inhibition of migrating lens epithelial cells at the capsular bend created by a sharp-edged intraocular lens after cataract surgery. Journal of cataract and refractive surgery 33: 1065–1070. 17531703 10.1016/j.jcrs.2007.02.022

[25] Nishi Y, Rabsilber TM, Limberger IJ, Reuland AJ, Auffarth GU (2007) Influence of 360-degree enhanced optic edge design of a hydrophilic acrylic intraocular lens on posterior capsule opacification. Journal of cataract and refractive surgery 33: 227–231. 17276262 10.1016/j.jcrs.2006.10.020

[26] Khandwala MA, Marjanovic B, Kotagiri AK, Teimory M (2007) Rate of posterior capsule opacification in eyes with the Akreos intraocular lens. Journal of cataract and refractive surgery 33: 1409–1413. 17662433 10.1016/j.jcrs.2007.04.022

[27] Abela-Formanek C, Amon M, Schauersberger J, Kruger A, Nepp J, et al (2002) Results of hydrophilic acrylic, hydrophobic acrylic, and silicone intraocular lenses in uveitic eyes with cataract: comparison to a control group. Journal of cataract and refractive surgery 28: 1141–1152. 12106722 10.1016/s0886-3350(02)01425-6

[28] Hayashi K, Hayashi H (2004) Posterior capsule opacification after implantation of a hydrogel intraocular lens. The British journal of ophthalmology 88: 182–185. 14736768 10.1136/bjo.2003.023580 PMC1771981

[29] Li N, Chen X, Zhang J, Zhou Y, Yao X, et al (2008) Effect of AcrySof versus silicone or polymethyl methacrylate intraocular lens on posterior capsule opacification. Ophthalmology 115: 830–838. 17964657 10.1016/j.ophtha.2007.06.037

[30] Nagata T, Minakata A, Watanabe I (1998) Adhesiveness of AcrySof to a collagen film. Journal of cataract and refractive surgery 24: 367–370. 9559473 10.1016/s0886-3350(98)80325-8

[31] Dorey MW, Brownstein S, Hill VE, Mathew B, Botton G, et al (2003) Proposed pathogenesis for the delayed postoperative opacification of the hydroview hydrogel intraocular lens. Am J Ophthalmol. 135: 591–598. 12719064 10.1016/s0002-9394(02)02154-2

[32] Hancox J, Spalton D, Cleary G, Boyce J, Nanavaty MA, et al (2008) Fellow eye comparison of posterior capsule opacification with AcrySof SN60AT and AF-1 YA-60BB blue-blocking intraocular lenses. J Cataract Refract Surg 34: 1489–1494. 18721708 10.1016/j.jcrs.2008.05.024

[33] Fujita S, Tanaka T, Miyata A, Hirose M, Usui M (2012) Cell adhesion and glistening formation in hybrid copolymer intraocular lenses. Ophthalmic Res 48: 102–108. 22517197 10.1159/000335981

[34] Aslam TM, Aspinall P, Dhillon B (2003) Posterior capsule morphology determinants of visual function. Graefe's archive for clinical and experimental ophthalmology = Albrecht von Graefes Archiv fur klinische und experimentelle Ophthalmologie. 241: 208–212. 10.1007/s00417-003-0626-8 12644945

[35] Biber JM, Sandoval HP, Trivedi RH, de Castro LE, French JW, et al (2009) Comparison of the incidence and visual significance of posterior capsule opacification between multifocal spherical, monofocal spherical, and monofocal aspheric intraocular lenses. Journal of cataract and refractive surgery 35: 1234–1238. 19545814 10.1016/j.jcrs.2009.03.013

[36] Sacu S, Menapace R, Wirtitsch M, Buehl W, Rainer G, et al (2004) Effect of anterior capsule polishing on fibrotic capsule opacification: three-year results. Journal of cataract and refractive surgery 30: 2322–2327. 15519082 10.1016/j.jcrs.2004.02.092

[37] Wren SM, Spalton DJ, Jose R, Boyce J, Heatley CJ (2005) Factors that influence the development of posterior capsule opacification with a polyacrylic intraocular lens. Am J Ophthalmol 139: 691–695. 15808166 10.1016/j.ajo.2004.12.013

