# Supplementary material for: Effect of Hydrophobic Acrylic versus Hydrophilic Acrylic Intraocular Lens on Posterior Capsule Opacification: Meta-Analysis
Source: PLoS One. 2013 Nov 5;8(11):e77864. doi: 10.1371/journal.pone.0077864 (PMC3818402; doi:10.1371/journal.pone.0077864)
Supplement: Prisma Flow Diagram S1 — PRISMA 2009 Flow Diagram. (DOC) [file pone.0077864.s002.doc]

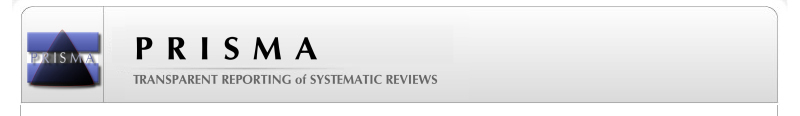
**PRISMA 2009 Flow Diagram**

**Screening**

**Included**

**Eligibility**

**Identification**

Records identified through database searching
(n =98 )

Additional records identified through other sources
(n =0 )

Records after duplicates removed
(n =1 )

Records screened
(n = 21)

Records excluded
(n = 76 )

Full-text articles assessed for eligibility
(n =9)

Full-text articles excluded, with reasons
(n = 12 )

Studies included in qualitative synthesis
(n =9 )

Studies included in quantitative synthesis (meta-analysis)
(n = 9)
